# Capillarized Liver Sinusoidal Endothelial Cells Undergo Partial Endothelial-Mesenchymal Transition to Actively Deposit Sinusoidal ECM in Liver Fibrosis

**DOI:** 10.3389/fcell.2021.671081

**Published:** 2021-07-05

**Authors:** Bai Ruan, Juan-Li Duan, Hao Xu, Kai-Shan Tao, Hua Han, Guo-Rui Dou, Lin Wang

**Affiliations:** ^1^Department of Hepatobiliary Surgery, Xi-Jing Hospital, Fourth Military Medical University, Xi’an, China; ^2^State Key Laboratory of Cancer Biology, Department of Biochemistry and Molecular Biology, Fourth Military Medical University, Xi’an, China; ^3^Department of Aviation Medicine, Center of Clinical Aerospace Medicine, Fourth Military Medical University, Xi’an, China; ^4^Department of Ophthalmology, Xi-Jing Hospital, Fourth Military Medical University, Xi’an, China

**Keywords:** liver fibrosis, extracellular matrix, myofibroblasts, endothelial cells, capillarization, endothelial-mesenchymal transition

## Abstract

Tissue-specific endothelial cells are more than simply a barrier lining capillaries and are proved to be capable of remarkable plasticity to become active collagen matrix-producing myofibroblasts (MFs) in solid organs with fibrosis. Liver sinusoidal endothelial cells (LSECs) also participate in the development of hepatic fibrosis, but the exact roles and underlying mechanism have been poorly understood in addition to capillarization. In this study, we demonstrate, by using single-cell RNA sequencing, lineage tracing, and colocalization analysis, that fibrotic LSECs undergo partial endothelial mesenchymal transition (EndMT) with a subset of LSECs acquiring an MF-like phenotype. These phenotypic changes make LSECs substantial producers of extracellular matrix (ECM) preferentially deposited in liver sinusoids but not septal/portal scars as demonstrated by immunofluorescence in animal models and patients with fibrosis/cirrhosis, likely due to their limited migration. Bioinformatic analysis verifies that LSECs undergo successive phenotypic transitions from capillarization to mesenchymal-like cells in liver fibrosis. Furthermore, blockade of LSEC capillarization by using YC-1, a selective eNOS-sGC activator, effectively attenuates liver damage and fibrogenesis as well as mesenchymal features of LSECs, suggesting that capillarization of LSECs might be upstream to their mesenchymal transition during fibrosis. In conclusion, we report that capillarized LSECs undergo a partial EndMT characterized by increased ECM production without activating cell mobility, leading to perisinusoidal ECM deposition that aggravate liver function and fibrogenesis. Targeting this transitional process may be of great value for antifibrotic treatment of liver fibrosis.

## Introduction

Liver fibrosis is characterized by abnormal extracellular matrix (ECM) deposition. Benefiting from elegant genetic tracing methodology, it is well-demonstrated that myofibroblasts (MFs) derived from activated hepatic stellate cells (HSCs), mesothelial cells, or portal fibroblasts (PFs) make major contribution to pathological ECM deposition (Li et al., 2013; Mederacke et al., 2013; Iwaisako et al., 2014; Lua et al., 2016; Kisseleva, 2017). However, alternative ECM sources have not been formally excluded, partly because other hepatic cell populations can transdifferentiate into MFs or transiently get the capacity of ECM synthesis in liver pathogenesis, depending on disease etiology and/or stages (Xu et al., 2014; Kisseleva, 2017). Recently, single-cell RNA sequencing (scRNA-seq) has emerged as a powerful tool to elicit transcriptomic changes in normal development and disease at the single-cell level. For liver fibrogenesis, it is demonstrated that hepatic MFs are heterogeneous and functionally diverse (Dobie et al., 2019; Krenkel et al., 2019). Other recent reports also depict the fibrogenic properties of endothelial cells (ECs) and macrophages in liver fibrosis (Ramachandran et al., 2019; Xiong et al., 2019). These corroborate the heterogeneity of various hepatic cell populations involved in ECM production and reveal their fate plasticity during fibrosis.

The liver is a highly vascular organ. Liver sinusoidal endothelial cells (LSECs), the specialized ECs featured by organized fenestrae and lack of a basement membrane, maintain hepatocyte homeostasis and orchestrate liver injury and repair (Poisson et al., 2017). During liver fibrogenesis, LSECs undergo dedifferentiation or capillarization by losing their fenestrae and developing a basement membrane, which unusually precedes the onset of fibrosis (DeLeve, 2015; Marrone et al., 2016). This leads to LSEC dysfunction contributing to hepatic fibrogenesis by facilitating HSC activation (Deleve et al., 2008; Xie et al., 2012) and hepatocyte reduction via disturbed angiocrine (Ding et al., 2014; Kostallari and Shah, 2016). On the contrary, blocking capillarization or restoration of differentiated phenotypes of capillarized LSECs is proved to promote fibrosis regression and prevent progression of cirrhosis (Xie et al., 2012). However, mechanisms coordinately controlling these phenotypic and functional alterations in LSECs in liver fibrosis remain incompletely characterized.

ECs from various solid organs are highly plastic in fibrotic diseases and are usually capable of transdifferentiating into collagen-producing MF-like cells through endothelial mesenchymal transition (EndMT) (Dejana et al., 2017; Piera-Velazquez and Jimenez, 2019). EndMT is a complex biological process in which ECs progressively evolve into cells with a mesenchymal phenotype, including displaying typical mesenchymal cell morphology, acquiring cellular motility, losing the expression of the EC-specific markers, and initiating the expression of mesenchymal cell–specific genes and the production of fibrillar collagens. It is generally accepted that activation of TGF-β signaling is the most potent triggering event for the induction of EndMT in fibrotic disorders. Numerous transcription factors, including Snai1, Slug, Zeb1, Zeb2, and Twist, are involved in this process although the detailed mechanisms have not been fully elucidated. However, given the complexity of the EndMT process, other as yet unidentified regulatory mechanisms are very likely involved in EndMT under specific conditions and cellular contexts (Piera-Velazquez and Jimenez, 2019). Early evidence (Rieder et al., 1987; Maher and McGuire, 1990) and previous reports (Dufton et al., 2017; Liu et al., 2017; Ribera et al., 2017) indicate, directly or indirectly, that LSECs are candidate cells depositing fibrogenic ECM in liver fibrosis, possibly through EndMT. However, several recent findings do not support the existence of EndMT of LSECs in liver fibrogenesis (Su et al., 2020; Terkelsen et al., 2020). Thus, further investigations are needed to shed more light on this debated matter. In this study, we show that, by using scRNA-seq, lineage tracing, and colocalization analysis in a mouse model of liver fibrosis, while a subset of LSECs become MFs, most LSECs undergo a phenotypic transition to form mesenchymal-like cells preceded by capillarization. The phenotypic change is reminiscent of partial EndMT and makes LSECs substantial producers of ECM preferentially in liver sinusoids.

## Materials and Methods

### Animals and Liver Fibrosis Models

Mice were maintained in a specific pathogen–free facility. The B6.129×1-Gt(ROSA)26Sor^tm1(EYFP)Cos^/J (Jackson Laboratory stock #006148, Bar Harbor, ME, MGI:2449038) mice were crossed with CDH5-CreERT mice (maintained in our laboratory). Tail DNA was used as templates to determine the genotypes of mice through polymerase chain reaction (PCR) analysis. Six-week-old male mice were injected intraperitoneally (i.p.) with tamoxifen (100 mg/kg, Sigma-Aldrich, St. Louis, MO) once a day for seven injections and used for further experiments 1 week after the last injection. To induce liver fibrosis, C57BL/6 or EC^YFP^ mice were injected i.p. with CCl_4_ (15% in olive oil, 0.6 μL/g body weight) twice a week for a total of 6 weeks with olive oil as control. Mice were sacrificed humanely 48 h after the last injection for further investigation. For establishment of bile duct ligation (BDL)-induced cholestatic fibrosis, male mice, 8–10 weeks old, were anesthetized and subjected to a midabdominal incision. The common bile duct was dissociated and ligated approximately 1 cm away from the porta hepatis. Age- and sex-matched littermates were selected for sham operation as normal control. Mice were sacrificed 3 weeks after operation for analyses. To assess the effect of YC-1 (Selleck, Houston, TX), a selective agonist of soluble guanylate cyclase (sGC) (Xie et al., 2012), on the mesenchymal phenotype of LSECs in a CCl_4_-fibrosis model, mice subjected to CCl_4_ were simultaneously given YC-1 (10 mg/kg, intragastric, daily, with DMSO as control) for a total of 3 weeks. All animal experiments were reviewed and approved by the Animal Experiment Administration Committee of the Fourth Military Medical University to ensure ethnical and humane treatment of animals.

### Human Biopsies

Human fibrotic liver biopsies were obtained from patients with hepatitis B virus–related end-stage liver cirrhosis or accepting liver transplantation in the Center for Transplantation, Xijing Hospital, Fourth Military Medical University (Supplementary Table 1). Human non-cirrhotic liver samples were collected from adjacent tumor tissue of patients with hepatic hemangioma at the time of hepatectomy surgery. All subjects signed informed consent for use of their samples in this study. The use of human samples was approved by the Ethics Committee of Xijing Hospital.

### Tissue Harvesting

At the indicated time points, mice were anesthetized by injection of 1% pentobarbital sodium i.p. Whole blood was collected through eyeball extraction. After clotting at room temperature for 2 h, serum samples were collected for further biochemistry measurement through centrifuging at 12,000 × g for 15 min. Mice were then perfused with PBS through the left ventricle. Liver lobes were removed, separated, and processed for subsequent experiments, including histology, immunofluorescence, and scanning electron microscope (SEM) observation.

For histology, freshly separated liver samples were fixed in 10% buffered formalin, embedded in paraffin, and sectioned to 10 μm thickness. Hematoxylin and eosin and Sirius red (Sigma-Aldrich, St. Louis, MO) staining was performed following standard procedures on paraffin-embedded sections.

### Immunostaining, Image Acquisition, and Analysis

To prepare liver cryosections for immunofluorescence analysis, mice were perfused with PBS and liver samples were fixed in 4% paraformaldehyde (PFA) for 2 h, washed with PBS, dehydrated in graded sucrose solutions at 4°C overnight, and snap frozen in optimum cutting temperature compound (Tissue-Tek, Sakura) at –80°C. For microscopy, mice liver cryosections (8 μm) were dried at room temperature for 2 h, followed by washing with PBS. Samples were blocked and permeabilized with QuickBlock^TM^ blocking buffer (Beyotime, Haimen, China) at room temperature for 1 h. Sections were then incubated with primary antibodies (listed in Supplementary Table 2) at 4°C overnight. After washing with PBS, sections were incubated with secondary fluorescent antibodies at room temperature for 2 h. Nuclei were counterstained with Hoechst 33342 (Invitrogen, Carlsbad, CA). Confocal imaging was performed on an Olympus FV1000 laser-scanning confocal fluorescence microscope (Olympus). To determine the fluorescence signals of colocalization of the sections, more than five random, high-power sinusoidal fields of each slide were captured and independently quantified by two investigators in a blinded fashion using Image-Pro Plus 6.0.

For cell immunofluorescence, cultured LSECs were grown on cover slides in 24-well plates for indicated days, fixed in 4% PFA for 15 min, washed with PBS, blocked and permeabilized with QuickBlock^TM^ blocking buffer (Beyotime) for immunostaining for 1 h at room temperature, followed by incubation of primary antibodies at 4°C overnight. On the second day, cells were washed and incubated with secondary fluorescent antibody at room temperature for 2 h. Hoechst 33342 (Invitrogen) was used to counterstain nuclei. Photographs were taken using fluorescence microscope (BX51, Olympus) or confocal microscope (FV1000, Olympus).

### Isolation and Culture of Mouse Liver Cells

LSECs (Duan et al., 2018) and HSCs (Chen et al., 2015; Mederacke et al., 2015) were isolated from mice by a two-step collagenase perfusion method as previously described with modifications. Briefly, mice were anesthetized with 1% pentobarbital sodium, perfused with 25 ml prewarmed Ca^2+^ and Mg^2+^-free, EDTA-containing buffer (9 g/L NaCl, 0.416 g/L KCl, 2.1 g/L NaHCO_3_, 1.08 g/L glucose, 4.8 g/L Hepes, 0.58 g/L EDTA) through the inferior vena cava for approximately 5 min with the portal vein severed for drainage. Then, the liver was digested by perfusion with another 25 ml prewarmed Ca^2+^-, Mg^2+^-, and type IV collagenase-containing buffer (9 g/L NaCl, 0.416 g/L KCl, 2.1 g/L NaHCO_3_, 1.08 g/L glucose, 4.8 g/L Hepes, 0.222 g/L CaCl_2_, 0.4065 g/L MgCl_2_⋅6H_2_O, 0.4 mg/ml collagenase IV) for 5 min. The liver was then removed, finely minced using gentle MACS C-tubes (Miltenyi Biotec, Bergisch Gladbach, Germany) and a tissue dissociator (Miltenyi) in 5 ml digestive perfusion buffer containing 100 μg/ml DNase I (Roche, Basel, Switzerland). After 30 min digestion with gentle shaking in a 37°C incubator, single cell suspension was obtained by passing through a 100-μm cell mesh. Hepatocytes were eliminated by three times of centrifugation at 50 × g for 3 min. Hepatic non-parenchymal cells (NPCs) were collected by centrifugation at 400 × g for 7 min, and the pelleted cells were resuspended in 4 ml 17.6% OptiPrep (Axis-Shield, Oslo, Norway). Then, 4 ml of 11.5% OptiPrep and 2 ml of DMEM were sequentially loaded on the top of the suspension. After centrifugation at 1,400 × g for 20 min without break, HSCs were obtained at the interface between the top and intermediate layer. The LSEC fraction was obtained at the interface between the bottom and intermediate layer and was purified using mouse LSEC-binding magnetic beads (Miltenyi) according to the manufacturer’s instructions.

For culturing, freshly isolated LSECs were plated in ECM supplemented with 5% fetal bovine serum, endothelial cell growth factor supplements, and 1% penicillin/streptomycin (ScienCell, San Diego, CA). After adhesion for 4 h, dishes were washed to remove debris and dead cells. Medium was replaced every 2 days. Cell samples were collected at the indicated days. FITC-labeled formaldehyde-treated serum albumin (FITC-FSA) was prepared as previously described (Seternes et al., 2002). For *in vitro* endocytosis assay, 100 μg/ml FITC-FSA and 20 μg/ml fluorescent acetylated low-density lipoprotein (Dil-ac-LDL, Solarbio, Beijing, China) were independently added into the LSEC medium. Cells were washed with PBS 10 min later, fixed, counterstained with Hoechst 33342, and imaged under a fluorescence microscope (BX51, Olympus). For *in vitro* activation of sGC, YC-1–sGC activator (30 μM, Selleck) were added into the medium with DMSO as control.

### SEM

For SEM analysis of liver tissues, mice were perfused with PBS to remove blood cells, followed by perfusion with a fixative (2.5% glutaraldehyde in 0.1 mol/L cacodylate buffer, pH7.4, 350 mOsm). The liver was then removed, cut into pieces, and immersed in 2.5% glutaraldehyde solution. For observation of *in vitro* cultured LSECs, cells were washed with PBS and fixed directly with 2.5% glutaraldehyde solution. Collected samples were then dehydrated in ethanol, dried in a vacuum desiccator, mounted on aluminum stabs, sputter-coated with gold, and viewed under an S-3400N scanning electron microscope (Hitachi, Tokyo, Japan).

### Flow Cytometry

Single cell suspension of LSECs was prepared as described. For surface staining, cells were incubated with indicated antibodies (listed in Supplementary Table 2) in flow cytometry staining buffer (eBioscience, San Diego, CA) for 30 min, followed by staining with fluorescent secondary antibody when necessary. For cytoplasmic staining, cells were preliminarily fixed for 10 min and permeabilized for 30 min using the intracellular fixation and permeabilization kit (eBioscience), followed by staining with antibodies in permeabilization buffer for another 30 min. Samples were analyzed with a FC500 flow cytometer (Beckman), and data were analyzed using the Flowjo 7.6 software. Unstained LSECs were used for determining gates, and isotype antibodies were used for negative control.

### Biochemistry

Serum samples were collected as described. The level of alanine aminotransferase (ALT) or aspartate aminotransferase (AST) was assessed to determine the degree of liver injury on an automatic biochemistry analyzer (Chemray240, Rayto, Shenzhen, China) using a kit (BioSino Bio-Technology & Science Inc., Beijing, China).

### Reverse Transcription (RT)-PCR

Total RNA was extracted from cells using the TRIzol reagent (Invitrogen) according to the manufacturer’s instructions. Quantification and quality evaluation of extracted total RNA was carried out by spectrophotometry using NanoDrop (Thermo Fisher Scientific, Waltham, MA). cDNA was synthesized from 2 μg total RNA by using a PrimeScrip RT reagent kit (Takara, Dalian, China). After reverse transcription, quantitative PCR was carried out with the SYBR Premix EX Taq^TM^ II kit (Takara) on an ABI 7500 real-time PCR system (Applied Biosystems, Foster City, CA) with β-actin as an internal control. Primers used are listed in Supplementary Table 3.

### Western Blotting

Freshly isolated cells were lysed in RIPA buffer (Beyotime) supplemented with 10 mM phenylmethanesulfonyl fluoride for 30 min on ice. Samples were then centrifuged at 12,000 rpm for 15 min at 4°C. Supernatants were collected and quantified by using a BCA protein assay kit (Solarbio) according to the manufacturer’s instructions. Next, 5 × reduced SDS-PAGE loading buffer (Beyotime) was added, and the protein samples were boiled for 5 min, followed by SDS-PAGE and Western blotting using antibodies listed in Supplementary Table 2, according to the standard protocols. The membranes were incubated with primary antibodies overnight at 4°C, washed with Tris-buffered saline with 0.1% Tween-20 (TBST, pH8.0) three times, and incubated with HRP-conjugated secondary antibodies for 1 h at room temperature. After three washes with TBST, the membranes were developed by using the enhanced chemiluminescence kit (Millipore). Images were captured by ChemiDoc^TM^ XRS + System (Bio-Rad, Hercules, CA) and analyzed by using Image Lab^TM^ Software (Bio-Rad).

### Transcriptome RNA-Sequencing and Bioinformatics Analysis

Bulk RNA-seq of purified LSECs from mice was performed by commercial service from Guangzhou RiboBio Co., Ltd., with Illumina HiSeq3000. Bioinformatic analysis of heat map illustration was carried out using the OmicShare tools, a free online platform for data analysis^1^. For gene set enrichment analysis (GSEA), gene set collections from the Molecular Signatures Database (MSigDB) 4.0^2^ were used.

### scRNA-seq

For scRNA-seq of hepatic NPCs from mice, cells were collected after density-gradient centrifugation. Briefly, liver was digested by the two-step collagenase perfusion method. Hepatocytes and debris were eliminated by repeated low-speed centrifugation and density-gradient centrifugation, respectively. Cells among the top and the intermediate layers were simultaneously collected, and cell viability was tested by trypan blue (>90%). Cell suspensions (∼10,000 cells) were then loaded on a chromium single cell instrument (10 × genomics) to generate single-cell GEMs. GEM-reverse transcriptions (GEM-RTs) were performed in a S1000 Touch Thermal Cycler (Bio-Rad). Then, scRNA-seq libraries were prepared using a Chromium Single-cell 3′ Library and Gel Bead Kit v3 (10 × genomics). The barcoded sequencing libraries were quantified by quantitative PCR using the KAPA Library Quantification Kit (KAPA Biosystems). Sequencing was performed on an Illumina Hiseq3000 to obtain a sequencing depth of ∼50,000 reads per cell (10 × genomics). Cell Ranger software (version 3.0.1) was used to convert raw BCL files to FASTQ files, alignment, and count quantification. Reads with low-quality barcodes and unique molecular identifiers (UMIs) were filtered out and then mapped to the mouse USCS mm10 reference genome. Reads uniquely mapped to the transcriptome and intersecting an exon at least 50% were considered for UMI counting. Before gene quantification, the UMI sequences were corrected for sequencing errors, and valid barcodes were then obtained. The cell-by-gene matrices were produced via UMI counting and cell barcode calling. Single cells were filtered for downstream analysis by the following criteria: mitochondria percentage of UMI count less than 10% of the total UMI count and number of detected genes more than 200. Gene expression (in UMI) is scale normalized and then transformed into log-space. Seurat suite version 3.0 was then used for downstream analysis (Stuart et al., 2019). For clustering, PCA was performed for dimension reduction. The top 10 principal components were selected by using a permutation-based test implemented in Seurat and passed to t-distributed stochastic neighbor embedding for clustering visualization. The identity for each cluster was assigned based on the prior knowledge of marker genes. A higher resolution parameter was applied for subclustering of the MF cluster. Cell doublets were tested and excluded by using Scrublet analysis^3^ to rule out possibilities of cell contamination (Wolock et al., 2019). Cloupe files were concurrently generated as input for a graphical user interface browser, Loupe Cell Browser 3.0.1 to present the clustering of cell population, individual tSNE plots for the given genes, and a heat map of differentially expressed genes among indicated cell types. Violin plots were generated by using GraphPad Prism 8.0.2. Pseudotemporal analysis was performed on a filtered subset of indicated clusters by using the Monocle R package (Trapnell et al., 2014). scRNA-seq in this study was supported by Genergy Inc., Shanghai, China.

### Statistics

Morphometric measurement of images was performed using Image-Pro Plus 6.0. Statistical analysis was carried out with the SPSS 12.0 program. Comparisons between two groups were undertaken using a two-tailed unpaired Student’s *t*-test as indicated. Data are presented as means ± SD. Differences with a *P-*value of less than 0.05 were considered statistically significant.

## Results

### A Subset of LSECs Undergo EndMT in Liver Fibrosis

**FIGURE 1:**
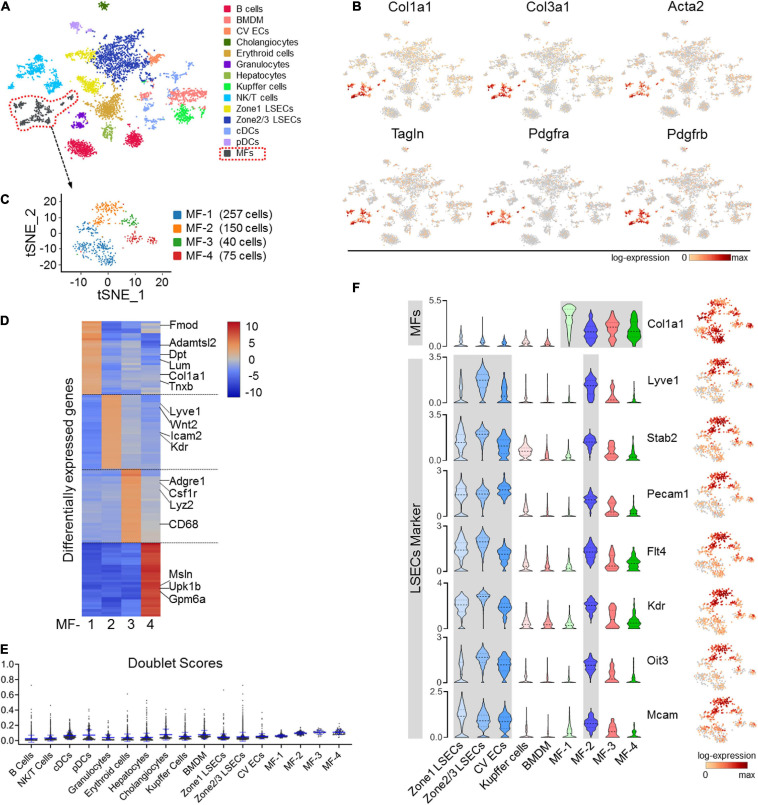
scRNA-seq of NPCs in CCl_4_-induced liver fibrosis. **(A)** Clustering of 6,437 cells (mean number of genes per cell = 1,768) from the liver NPCs of a CCl_4_-treated male mouse. **(B)** The expression of representative MF marker genes, including Col1a1, Col3a1, Acta2 (α-SMA), Tagln (SM22), Pdgfra, and Pdgfrb, in the population (marked with dotted lines in red) was visualized by gene tSNE. **(C)** The MF population in (A) was further clustered into four subsets and visualized by gene tSNE. **(D)** The top 30 differentially expressed genes in the four MF subpopulations are illustrated by heat map. **(E)** Cell doublet scores of the fibrotic-NPCs per cluster. **(F)** Individual gene tSNE and violin plots are used to show the expression of MF marker Col1a1 and LSEC markers Lyve1, Stab2, Pecam1, Flt4, Kdr, Oit3, and Mcam across different cell types. The *y*-axis is the log-scale normalized read count.

To access cellular alterations in fibrosis, we performed scRNA-seq of liver NPCs collected from fibrotic (CCl_4_-insulted) and control mice. In the normal control, only small populations of ECM-expressing cells were identified, including quiescent HSCs, PFs, and mesothelial cells (but not ECs), which barely expressed the activated MF marker α-SMA (Acta2) (Supplementary Figures 1A–C). In fibrotic liver, in contrast, a significant MF cluster expressing canonical mesenchymal and ECM-related genes, including Col1a1, Col3a1, α-SMA, SM22 (Tagln), Pdgfra, and Pdgfrb, was identified in 14 clusters from 6,437 cells (Figures 1A,B and Supplementary Figure 2A). Further analysis of this fibrotic MF cluster revealed four subtypes with distinct signatures (Figure 1C). While MF-1 and MF-4 express typical ECM organization genes and mesothelial markers, respectively, MF-3 expresses macrophage markers, reminiscent of macrophage-MF transition in fibrosis (Wang et al., 2017; Haider et al., 2019) (Figure 1D and Supplementary Figure 2B). MF-2, which constitute 28.7% (150/522 cells) of the MF population, expressed genes featured in LSECs, such as Lyve-1, Stab2, Pecam1, Flt4, Kdr, Oit3, and Mcam (Figures 1C,F). To be noted, doublet-likelihood scores were low across clusters, illustrating that the multifeatures of MF subpopulations were not because of cell doublets or contaminations (Figure 1E). These results corroborate the heterogeneous origin of MFs as determined in recently published single-cell studies (Dobie et al., 2019; Krenkel et al., 2019) and suggest that LSECs contribute a specific MF subtype in liver fibrosis as previously reported (Ribera et al., 2017).

### Validation of LSECs EndMT by Lineage Tracing

**FIGURE 2:**
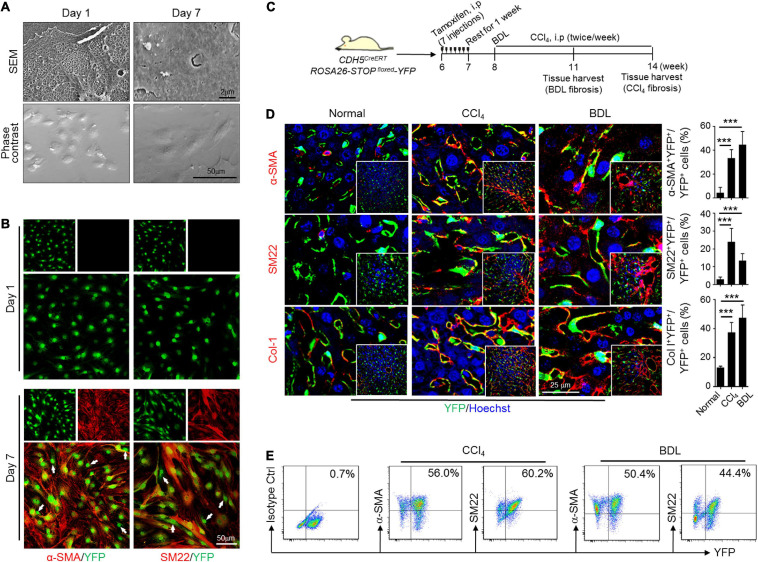
Lineage tracing study verifies EndMT of LSECs *in vitro* and *in vivo*. **(A,B)** LSECs from genetically labeled EC^YFP^ mice were cultured for indicated days, observed under an SEM (top) or a light microscope (phase contrast, bottom) and further counterstained with anti-α-SMA and anti-SM22 (*n* = 3). Arrows indicate LSECs that are negative for α-SMA or SM22. **(C)** Strategy for establishing liver fibrosis models on EC^YFP^ mice. **(D)** Liver cryosections from EC^YFP^ mice subjected to CCl_4_- or BDL-fibrosis were stained for YFP plus α-SMA, SM22, or Col-1. The percentages of YFP^+^α-SMA^+^, YFP^+^SM22^+^, and YFP^+^Col-1^+^ cells in total YFP^+^ LSECs were quantitatively compared with the normal control (*n* = 4 for the CCl_4_ model and *n* = 5 for the BDL model). Bars = means ± SD; ***P < 0.001 vs. normal control, using two-tailed *t*-test. **(E)** LSECs were isolated from mice in (C), and analyzed by FACS for endogenous YFP and α-SMA/SM22 after cytoplasmic anti-α-SMA/anti-SM22 staining.

To further testify the phenotypic transition of LSECs, EC^YFP^ (CDH5^CreERT^-ROSA26-STOP^floxed^-YFP) mice were bred to label ECs specifically with YFP by tamoxifen induction. Prominent colocalization of LSEC marker Lyve1 with YFP proved the efficiency of CDH5-driven labeling (Supplementary Figure 3A). Moreover, HSC marker Desmin and Kupffer marker F4/80 could be finely discriminated from endothelial YFP, indicating a satisfactory specificity and resolution of our colocalization detection (Supplementary Figures 3B,C). Then, we isolated, identified and cultured LSECs from wild-type or EC^YFP^ mice to examine their phenotypic transitions *in vitro* (Supplementary Figures 4A–D). After extended culture for 7 days, LSECs not only underwent capillarization, but also exhibited spindle-shaped and elongated cell morphology and remarkably increased expression of α-SMA, SM22, and Col-1, suggesting mesenchymal transition (Figures 2A,B and Supplementary Figures 4E–G). Meanwhile, unchanged HSC markers (Lrat, Reln, and Desmin) examined by qPCR, positive expression of VE-cadherin, and negatively immunostained Desmin by immunofluorescence demonstrate that the observed mesenchymal features of *in vitro* cultured LSECs were not because of HSC contaminations or a full cell fate transition to HSCs (Supplementary Figures 4E,G). Thus, these results uncovered an unanticipated property of plasticity of LSECs, similar to the activation process of quiescent HSCs to activated MFs during culturing. EC^YFP^ mice were then subjected to CCl_4_- or BDL-fibrosis (Figure 2C and Supplementary Figure 5). Colocalization analysis of YFP and mesenchymal markers showed that ∼50% of YFP-labeled LSECs co-expressed α-SMA, SM22, and Col-1 in CCl_4_- or BDL-induced fibrotic liver but not in the controls (Figure 2D), which was reconfirmed by labeling LSECs with Isolectin B4 (Supplementary Figure 6). LSECs from the fibrotic EC^YFP^ mice were further isolated and analyzed by FACS. The result similarly demonstrates a considerable proportion of YFP^+^ LSECs co-expressing α-SMA and SM22 in cytoplasm (Figure 2E). Thus, these data collectively verify that LSECs could undergo an EndMT-like transdifferentiation both *in vitro* and *in vivo*.

### Mesenchymal Transition of LSECs Is Partial in Liver Fibrosis

**FIGURE 3:**
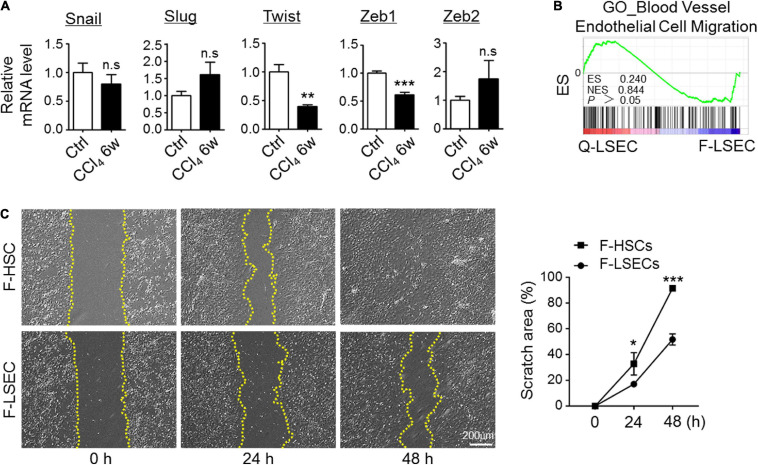
Mesenchymal transition of LSECs is partial in fibrosis. **(A)** LSECs were isolated from control and fibrotic mice, and the expression of EndMT-related transcription factors was determined by qRT-PCR (*n* = 3–4). Bars = means ± SD; **P < 0.01, ***P < 0.001 vs. control LSECs, using two-tailed *t*-test. **(B)** Genes reflecting vascular endothelial cell migration were compared between Q-LSEC and F-LSEC with GSEA (*n* = 3). **(C)** LSECs and HSCs were isolated from the fibrotic mice (F-LSEC and F-HSC, respectively), and cell migration was determined by scratch assay (*n* = 4). Bars = means ± SD; *P < 0.05, ***P < 0.001 vs. fibrotic-LSECs, using two-tailed *t*-test; n.s., not significant. Q-LSEC, quiescent LSEC; F-LSEC, fibrotic LSEC; F-HSC, fibrotic HSCs; GSEA, gene set enrichment analysis.

Because EndMT is usually initiated by induction of the specific transcription factors, such as Snail, Slug, Twist, Zeb1, and Zeb2 (Piera-Velazquez and Jimenez, 2019), we then isolated LSECs from the control and CCl_4_-injured mice and compared the expression level of these EndMT-inducers by RT-PCR. As a result, we did not detect significant upregulation of typical EndMT-related transcription factors in fibrotic LSECs (Figure 3A). Then, we tried to assess whether mesenchymal-transited LSECs acquired an increased capability of migration. We performed bulk-RNA sequencing on isolated LSECs. However, gene signatures reflecting EC-migration were insignificantly enriched between control and fibrotic LSECs as demonstrated by GSEA (Figure 3B). Then, the migration of LSECs and HSCs isolated from the fibrotic mice were compared by scratch assays, and the result showed that fibrotic LSECs displayed a much lower capacity to migrate in culture (Figure 3C). These results suggest that, following chronic liver injury, LSECs undergo a partial EndMT (Li et al., 2018; Piera-Velazquez and Jimenez, 2019) to form an intermediate phenotype of mesenchymal-like LSECs that produce a high amount of ECM with low migrating capacity.

### Partially Mesenchymal-Transited LSECs Contribute to Perisinusoidal ECM Deposition

**FIGURE 4:**
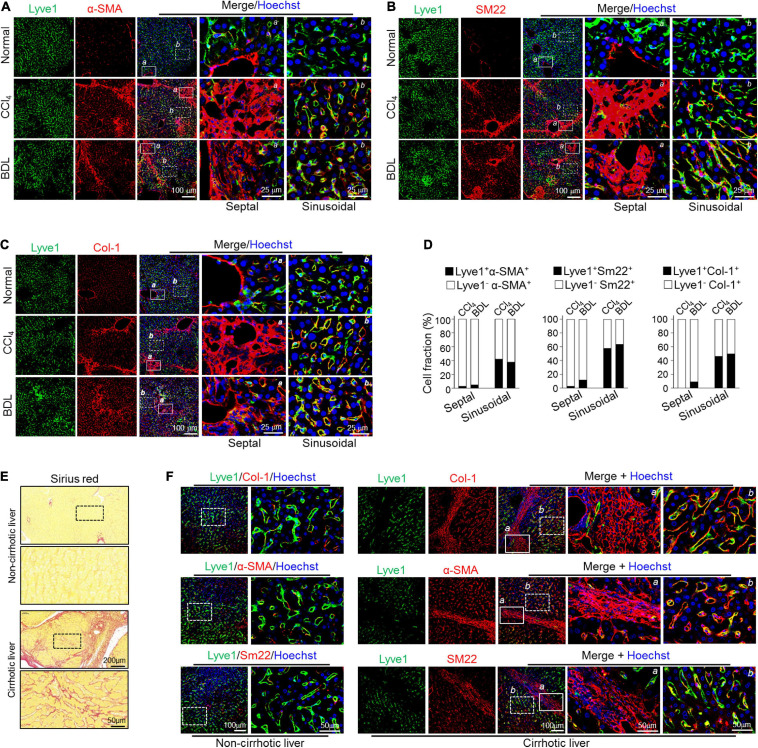
Mesenchymal-transited LSECs mainly contribute to sinusoidal but not septal/portal ECM deposition in liver fibrogenesis. **(A–C)** Mice were subjected to CCl_4_- or BDL-induced liver fibrosis (*n* = 3–4). Liver cryosections were stained for Lyve1 plus α-SMA (A), SM22 (B), or Col-1 (C) with nuclei counterstained with Hoechst. Representative septal/portal areas (*a*, box with solid lines) and sinusoidal areas (*b*, box with dotted lines) are shown at higher magnification. **(D)** Cell fractions of Lyve1^+^ mesenchymal (α-SMA^+^/SM22^+^/Col-1^+^) cells between the septal and the sinusoidal areas are shown. **(E)** Representative Sirius red staining showing sinusoidal fibrogenesis in human cirrhotic liver sections (*n* = 3 for healthy liver and *n* = 8 for cirrhotic liver). **(F)** Human non-cirrhotic and cirrhotic liver sections were stained for Lyve1 and MF markers (Col-1, α-SMA, and SM22). Representative septal areas (*a*, box with solid lines) and sinusoidal areas (*b*, box with dotted lines) are shown at higher magnification.

Because fibrotic LSECs exhibit limited migrating capacity compared with fibrotic HSCs, they likely deposit fibrotic ECM preferentially into liver sinusoids. Therefore, we immunostained LSEC marker Lyve1 and MF markers α-SMA/Sm22 in fibrotic liver sections. The result showed that strong colocalization of Lyve-1 and α-SMA/Sm22 was preferentially detected in the sinusoidal but barely in the septal regions (bridging-fibrosis regions) in both CCl_4_- and BDL-induced fibrosis models (Figures 4A,B,D). Consistently, the fibrotic liver also manifested significantly increased colocalization of Col-1, the major ECM component in fibrosis, and EC markers Lyve1 in sinusoidal regions (Figures 4C,D). In line with these, a distinguished fibrous ECM accumulation, as determined by Sirius red staining, could be obviously observed in the sinusoidal areas of CCl_4_- and BDL-mediated liver fibrosis (Supplementary Figure 5). In human cirrhotic livers, Sirius red examination also showed noticeable sinusoidal fibrosis in the liver parenchyma (Figure 4E), accompanied by strong colocalization of Lyve1 and MF markers in sinusoidal regions but not fibrotic septal regions (Figure 4F). These data collectively suggest that LSECs undergoing partial mesenchymal transition actively deposit ECM primarily in sinusoidal areas in liver fibrosis.

### Successive Phenotypic Transition of LSECs From Capillarization to EndMT in Liver Fibrosis

**FIGURE 5:**
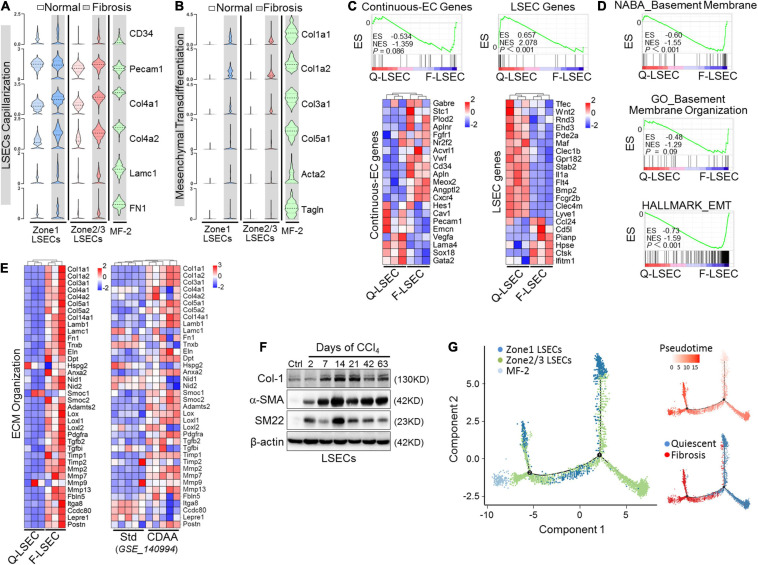
Capillarized LSECs undergo EndMT during liver fibrogenesis. **(A,B)** The expressions of capillarization markers (A) and mesenchymal markers (B) in different EC subpopulations between normal and fibrosis are shown by violin plots. **(C–E)** LSECs were isolated from mice injected with CCl_4_ or oil for 6 weeks and subjected to RNA-seq (*n* = 3). Continuous EC- and LSEC-associated genes were compared between the two types of LSECs with GSEA and heat maps (C). Transcriptomes were further analyzed by GSEA for vascular basement membrane formation/organization and epithelial mesenchymal transition (D). Genes involved in ECM organization were compared between normal and fibrotic LSECs (left, Q-LSEC vs. F-LSEC; right, Std-LSEC vs. CDAA-LSEC) with a heat map (E). **(F)** LSECs were isolated from wild-type mice treated with oil or CCl_4_ for the indicated days, and the expression of mesenchymal markers (Col-1, α-SMA, and SM22) was determined by Western blotting. **(G)** Pseudotemporal cell ordering of MF-2 and LSEC subpopulations along differentiation trajectories by using Monocle. Pseudotime is depicted from light to dark red. Q-LSEC, quiescent LSEC; F-LSEC, fibrotic LSEC; Std, standard; CDAA, choline-deficient, l-amino acid-defined; GSEA, Gene Set Enrichment Analysis.

Capillarization is the hallmark dysregulated phenotype of LSECs in liver fibrosis. To explore the in-depth relationships between capillarization and EndMT of LSECs during fibrogenesis, we analyzed our scRNA-seq and bulk RNA-seq of isolated-LSECs on these two pathological changes. In fibrotic liver, LSEC clusters upregulated genes of continuous ECs and basement membrane, including CD34, CD31 (Pecam1), Col4a1, Col4a2, LamC1, and FN1, and downregulated LSEC-associated genes, consistent with dedifferentiation or capillarization of LSECs (Geraud et al., 2017; Winkler et al., 2021; Figures 5A,C). Furthermore, we found that fibrotic LSECs also expressed other mesenchymal or ECM-related genes and were enriched for a hallmark gene set of epithelial-mesenchymal transition following capillarization (Figures 5B,D,E). The acquisition of mesenchymal features of fibrotic LSECs was validated as well by a recently published microarray data of Std- vs. CDAA-LSECs (Winkler et al., 2021), in which NASH-related perisinusoidal fibrosis could be finely modeled without the tendency to turn into progressive bridging or peri-portal fibrosis (Figure 5E). Western blotting also verified that the expression of α-SMA, SM22, and Col-1 was upregulated in fibrotic LSECs on different days of CCl_4_ induction (Figure 5F). More importantly, pseudotime analysis showed a successive transition of quiescent LSECs into fibrotic Col-1-expressing, mesenchymal-like LSECs, and MF-like cells (MF-2) (Figure 5G). These suggest that LSECs undergo successive phenotypic transitions, i.e., from differentiated LSECs to capillarization and then to mesenchymal, with a small population converted to MFs, in liver fibrosis.

### Blocking Capillarization Abolishes LSECs EndMT

**FIGURE 6:**
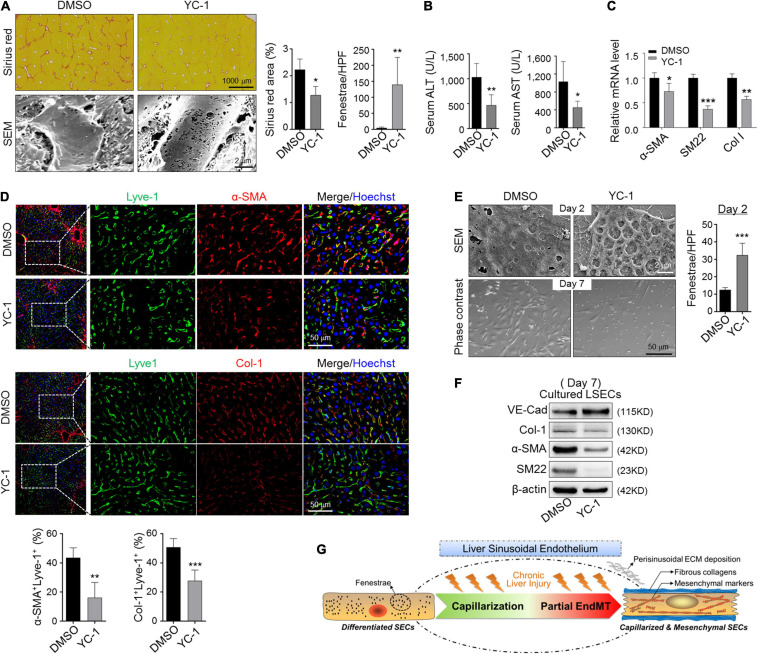
Blocking capillarization simultaneously reverses the mesenchymal phenotypes of LSECs during liver fibrosis and *in vitro* culturing. **(A,B)** Mice bearing CCl_4_-fibrosis were treated with DMSO or YC-1. Liver sections were stained by Sirius red or observed under SEM. Sirius red^+^ areas and fenestrae per high power field were quantitatively compared between the two groups (A, *n* = 6). Serum ALT and AST were determined (B, *n* = 6). **(C)** LSECs were isolated from mice in (A) and the expression of α-SMA, SM22, and Col-1 was evaluated by qRT-PCR (*n* = 4). **(D)** Colocalization of Lyve1 and α-SMA/Col-1 in the sinusoidal areas is shown and quantitatively compared (*n* = 4). **(E,F)** LSECs were cultured in the presence of DMSO or YC-1, and were observed under SEM and light microscope on days 2 and 7 of the culture, respectively. Fenestrae were quantified on day 2 and compared (E, *n* = 4). Expression of VE-cadherin and mesenchymal markers α-SMA, SM22, and Col-1 on day 7 was determined by Western blotting (F). Bars = means ± SD; *P < 0.05, **P < 0.01, ***P < 0.001 vs. DMSO, using two-tailed *t*-test for (A–E). **(G)** Cartoon image shows that capillarized LSECs undergo partial endothelial-mesenchymal transition to actively deposit sinusoidal ECM in liver fibrosis.

Because eNOS-sGC is documented to modulate capillarization of LSECs in both steady and fibrotic states (Xie et al., 2012; Duan et al., 2018), we access whether eNOS-sGC is involved in EndMT. We treated CCl_4_-induced fibrotic mice with YC-1, an activator of sGC. Injection of YC-1 increased LSEC fenestration and reduced fibrosis and attenuated liver damage (Figures 6A,B), consistent with previous reports (Xie et al., 2012). Then, LSECs were purified from fibrotic mice and evaluated by RT-PCR for mesenchymal gene expression. The results showed decreased α-SMA, SM22, and Col-1 in fibrotic LSECs treated with YC-1 (Figure 6C). Consistently, we found the colocalization of α-SMA/Col-1 in LSECs was repressed by YC-1 injection during CCl_4_ fibrosis, suggesting diminished sinusoidal ECM deposition (Figure 6D). In addition, treatment of *in vitro* cultured LSECs with YC-1 increased fenestration and blunted the mesenchymal morphology (Figure 6E). Western blotting indicated that YC-1 treatment upregulated endothelial marker VE-cad and repressed mesenchymal markers Col-1, α-SMA, and SM22 (Figure 6F). These collectively imply that blocking capillarization of fibrotic-LSECs simultaneously prevents the acquisition of mesenchymal features of themselves.

## Discussion

As demonstrated by previous genetic tracing experiments, HSCs, PFs, and mesothelial cells are dominant precursors of hepatic MFs (Li et al., 2013; Mederacke et al., 2013; Iwaisako et al., 2014; Lua et al., 2016; Kisseleva, 2017). However, most, if not all, commonly used Col I reporters in tracing experiments employ cloned enhancer fragments of *pro-α1(I)* (Yata et al., 2003) or *pro-α2(I)* (De Val et al., 2002) genes, which might exclude visualization of Col-1 expression driven by genomic elements outside these enhancers. Thus, alternative ECM sources have never been formally ruled out, partly because other hepatic cell populations, such as bone marrow–derived cells (Kisseleva et al., 2006), hepatocytes (Zeisberg M. et al., 2007; Oh et al., 2018), or hepatic ECs (Dufton et al., 2017; Ribera et al., 2017) may transiently acquire mesenchymal phenotype and get the capacity of ECM synthesis in the context of liver fibrogenesis, depending on disease etiology, stage, and hepatic architecture although they seldom join in the MF pool (Taura et al., 2016; Kisseleva, 2017). In fact, indirect evidence implies the fibrogenic role of LSECs as potential pathological matrix-depositing cells (Rieder et al., 1987; Maher and McGuire, 1990; Liu et al., 2017; Ribera et al., 2017), but their concrete contribution to the pathogenesis of liver fibrosis as well as the mechanisms regulating their phenotypic changes have never been completely characterized.

ScRNA-seq recently emerges as a powerful approach to elicit transcriptomic changes in normal development and disease at unprecedented resolution (Ramachandran et al., 2020). For liver fibrogenesis, Dobie et al. (2019) dissect hepatic mesenchyme in healthy and CCl_4_-induced fibrotic liver at the single-cell level and identify different mesenchymal cell types and functional zonation of collagen-producing MFs in hepatic Pdgfrb^+^ cells. However, they exclude Icam2^+^ (an LSEC marker) cells through FACS sorting at the initial step, which may rule out the possibilities of LSEC-originated MFs in their system. A parallel study accomplished by the same group identifies seven endothelial subpopulations in human cirrhotic liver, and one of the fibrotic niche–constituting subpopulations, SAEndo(1), expresses ECM organization-related genes (Ramachandran et al., 2019). In addition, data sets obtained from the human livers indeed manifest a sharp decrease of LSEC population in the cirrhotic samples. Thus, it seems difficult to discriminate whether the absence of LSEC-EndMT in their data is due to a loss of cirrhotic-LSEC information or a fully transdifferentiated process that might have already taken place during cirrhosis as similarly postulated by Winkler et al. (2021). Other recent single-cell studies also depict the variable properties of hepatocytes (Chang et al., 2019), HSCs (Krenkel et al., 2019), and macrophages (Xiong et al., 2019; Omori et al., 2020) in liver fibrosis. These data collectively corroborate the heterogeneity of various hepatic cell populations involved in ECM deposition and reveal their fate plasticity during liver fibrosis. Our scRNA-seq analysis of physically isolated liver NPCs identifies four MF subpopulations. Two of them, MF-1 and MF-4, are likely derived from HSCs and mesothelial cells, respectively, and MF-3 displays a macrophage-like signature reminiscent of the possibilities of macrophage-mesenchymal transition in liver fibrosis (Krenkel et al., 2019) and other fibrotic diseases (Wang et al., 2017; Haider et al., 2019). Of note, MF-2 express endothelial markers, suggesting that they are likely derived from ECs by EndMT, similar to fibrogenesis in other solid organs, such as the heart (Zeisberg E. M. et al., 2007), kidney (Zeisberg et al., 2008), and lung (Hashimoto et al., 2010). Our data confirms Ribera et al. (2017) who report a small proportion of liver ECs undergoing EndMT in fibrosis. Moreover, both scRNA-seq and genetic tracing as well as immunofluorescence and Western blotting indicate that a considerable proportion of LSECs turn to produce ECMs in liver fibrosis. It is well-known that LSECs undergo capillarization in fibrosis (DeLeve, 2015; Marrone et al., 2016; Poisson et al., 2017). Our data suggest that chronic liver injury could drive LSECs to further gain a mesenchymal signature and secret pathological ECM (Figure 6G). This phenotypic transition of LSECs is partial, by which they co-express molecular markers of endothelial and mesenchymal cells and gain a significant capacity of ECM secretion but lack migration during fibrogenesis, indicating an intermediate EndMT phenotype (Piera-Velazquez and Jimenez, 2019). Whether the fibrotic-LSECs could fully transdifferentiate into HSC-originated MFs *in vivo* remains an interesting and challenging issue to be addressed. More importantly, blocking capillarization by a specific sGC activator (YC-1) abrogates mesenchymal transition of LSECs, suggesting that capillarization of LSECs is upstream to their mesenchymal transition during fibrosis. Therefore, although capillarization turns LSECs into capillary-like LSECs generating a basement membrane, these LSECs could become pathological ECM producers upon partial EndMT in liver fibrosis. However, one unresolved question in our study is that the concrete contribution of these partially transited LSECs to the population of active ECM-depositing cells as well as to the extent of peri-sinusoidal collagen matrix accumulation in the fibrotic livers remains incompletely characterized. Previous animal studies demonstrate that 27–35%, 30–50%, and ∼16% of MFs are from EC origin in the heart (Zeisberg E. M. et al., 2007), kidney (Zeisberg et al., 2008), and lung (Hashimoto et al., 2010) fibrosis models, respectively. However, conflicting results concerning contribution of EndMT to organ fibrosis still exist, mainly because of the methodological limitations of the lineage tracing of EndMT with satisfactory sensitivity and specificity (Frangogiannis, 2020). Regarding liver fibrosis, although Ribera et al. (2017) demonstrate the emergence of EndMT of LSECs in liver fibrosis, they conclusively state that the proportion of EndMT as well as their contribution to liver fibrogenesis is relatively small. Future studies using mouse models that allow precise cell fate mapping and targeted clearance of cellular sources of LSECs undergoing EndMT is more informative.

As described above, evidence in the past decade have already indicated that fully differentiated endothelium in adult organs is capable of remarkable plasticity to undergo EndMT, thereby contributing to pathogenesis of numerous human fibrotic disorders. It has been generally accepted that this mesenchymal-transition of ECs is, in most cases, an intermediate phenotype rather than a permanent shift, making this transitional process a novel and valuable target for antifibrotic therapies (Dejana et al., 2017; Piera-Velazquez and Jimenez, 2019). The liver is a highly vascularized organ in which LSECs, the specialized endothelium featured by organized fenestrae and lack of a basement membrane forming the wall of liver sinusoids, account for about 15–20% of hepatic cells (Poisson et al., 2017). In the event of chronic liver injury, capillarized LSECs play pivotal roles in initiating and aggravating liver fibrosis by multiple mechanisms, including impaired sinusoidal microcirculation, hepatocyte metabolism (Hammoutene and Rautou, 2019), HSC activation (Deleve et al., 2008; Xie et al., 2012), and abnormal angiocrine with reduced expression of hepatocyte mitogens, such as HGF and Wnt2a (Rafii et al., 2016; Duan et al., 2018). Mesenchymal transition might strengthen at least some of these roles of LSECs by increased secretion of fibrotic ECM. Du and colleagues highlight ECM-related molecules, which could be derived from mesenchymal LSECs as shown in this study, induce HSC activation via collagen condensation and fibrosis progression via fibrous matrix-mediated myofibroblast–fibroblast crosstalk called paratensile signaling (Liu et al., 2017, 2020). Moreover, increased ECM deposition may further disturb sinusoidal microcirculation, which could disrupt angiocrine signals for hepatocyte survival by β1 integrin-mediated mechanosensing (Lorenz et al., 2018). The functional consequences of these alterations are increased hepatocyte death and reduced hepatocyte regeneration, which erode liver function, leading to liver failure. Thus, it can be conceived that diminishing LSEC-derived perisinusoidal ECM deposition would convey considerable benefits for retarding liver fibrogenesis. More efforts need to be taken to unravel the detailed mechanisms regulating LSEC plasticity to the development of antifibrotic therapy in future studies. However, it should be noted that, sometimes, activation of a matrix-preserving fibrogenic program reflects a protective and reparative response, especially in acute tissue injuries, aiming at preserving the basic structural characteristics of the organ, thus preventing a catastrophic outcome (Frangogiannis, 2020). Moreover, MFs are believed to be master regulators of the regenerative process. On the one hand, MFs are an important source of trophic factors for hepatocytes in addition to matrix-producing cells during chronic liver injuries. MF depletion has been demonstrated to impair liver regeneration in adults (Kalinichenko et al., 2003). On the other hand, myofibroblastic cells can function as progenitors to regenerate murine livers in PHx- and BDL-injured models (Michelotti et al., 2013; Swiderska-Syn et al., 2014). Thus, in addition to active deposition of perisinusoidal ECM, whether the partially mesenchymal-transited LSECs exert a protective function on the injured hepatocytes or act as potential progenitors to differentiate into liver epithelial cells in the context of fibrosis should be further validated.

## Data Availability Statement

The original data of our bulk RNA-seq and scRNA-seq have been submitted to GEO database (https://www.ncbi.nlm.nih.gov/geo/) and can be accessed through the accession numbers: GSE120283 and GSE134037, respectively.

## Ethics Statement

The use of human samples was approved by the Ethics Committee of Xi-Jing Hospital, Fourth Military Medical University. The patients/participants provided their written informed consent to participate in this study. The animal study was reviewed and approved by the animal Ethics Committee of Fourth Military Medical University.

## Author Contributions

BR and J-LD performed most of the experiments and analyzed the data. HX assisted in the animal experiments and histological analysis. K-ST helped with the human sample collections, provided equipment, and offered valuable discussion. G-RD helped in the data collection and bioinformatics analysis. LW, HH, and G-RD fulfilled the experimental design and supervised the study. LW and HH wrote the manuscript. All authors contributed to the article and approved the submitted version.

## Conflict of Interest

The authors declare that the research was conducted in the absence of any commercial or financial relationships that could be construed as a potential conflict of interest.

## Footnotes

1 www.omicshare.com/tools

2 www.broadinstitute.org/gsea/msigdb/

3 github.com/AllonKleinLab/scrublet
