# Supplementary material for: Capillarized Liver Sinusoidal Endothelial Cells Undergo Partial Endothelial-Mesenchymal Transition to Actively Deposit Sinusoidal ECM in Liver Fibrosis
Source: Front Cell Dev Biol. 2021 Jul 5;9:671081. doi: 10.3389/fcell.2021.671081 (PMC8285099; doi:10.3389/fcell.2021.671081)
Supplement: Supplementary file 1 [file Data_Sheet_1.docx]

Supplementary Material

## Supplementary Figures
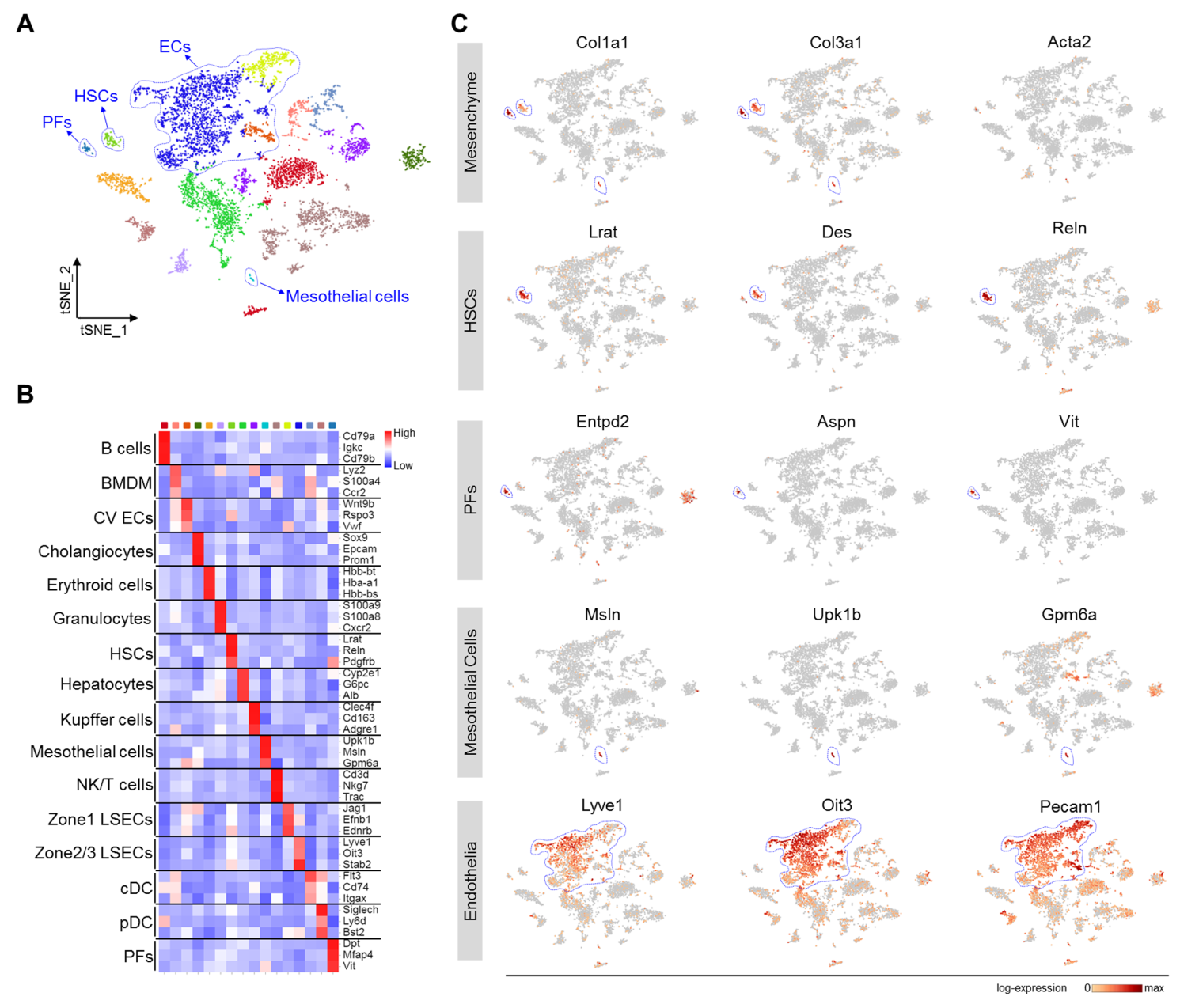


**Supplementary Figure 1.** Single-cell analysis of liver NPCs from the control mouse. **(A)** Clustering of 8130 cells (mean number of genes per cell = 1678) from the liver NPCs of a normal C57BL/6 male mouse revealed 16 cell types according to their gene expression profiles (upper). **(B)** Gene-expression heatmap of the representative marker genes for each cluster of the normal liver NPCs. Color scale: red, high expression; blue, low expression. **(C)** The proposed matrix-expressing (Col1a1 and Col3a1) cell populations were identified as HSCs, PFs and mesothelial cells but not hepatic ECs (marked with blue dotted line in *[A]* respectively), with their representative marker genes (Lrat, Des, and Reln for HSCs; Entpd2, Aspn and Vit for PFs; Msln, Upk1b and Gpm6a for mesothelial cells; Lyve1, Oit3 and Pecam1 for hepatic ECs) visualized by tSNE.


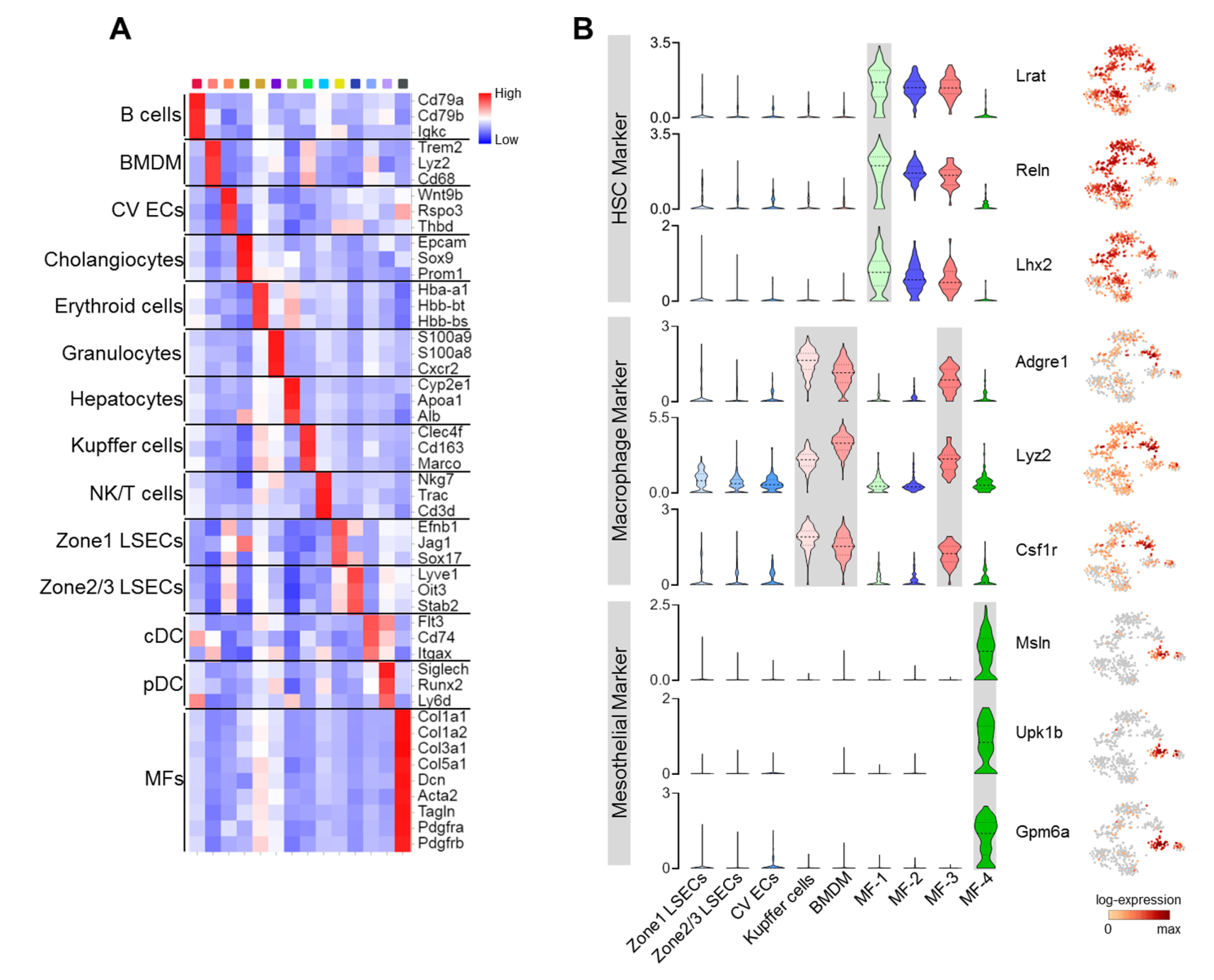


**Supplementary Figure 2.** Clustering and gene expression profile of the fibrotic liver NPCs. **(A)** Gene-expression heatmap of the representative marker genes for each cluster of the fibrotic liver NPCs. Color scale: red, high expression; blue, low expression. **(B)** The expression of markers for HSCs (Lrat, Reln, Lhx2), macrophages (Adgre1, Lyz2, Csf1r) and mesothelial cells (Msln, Upk1b, Gpm6a) in the indicated cell types was shown by violin plots and gene tSNE. The y axis is log-scale normalized read counts.


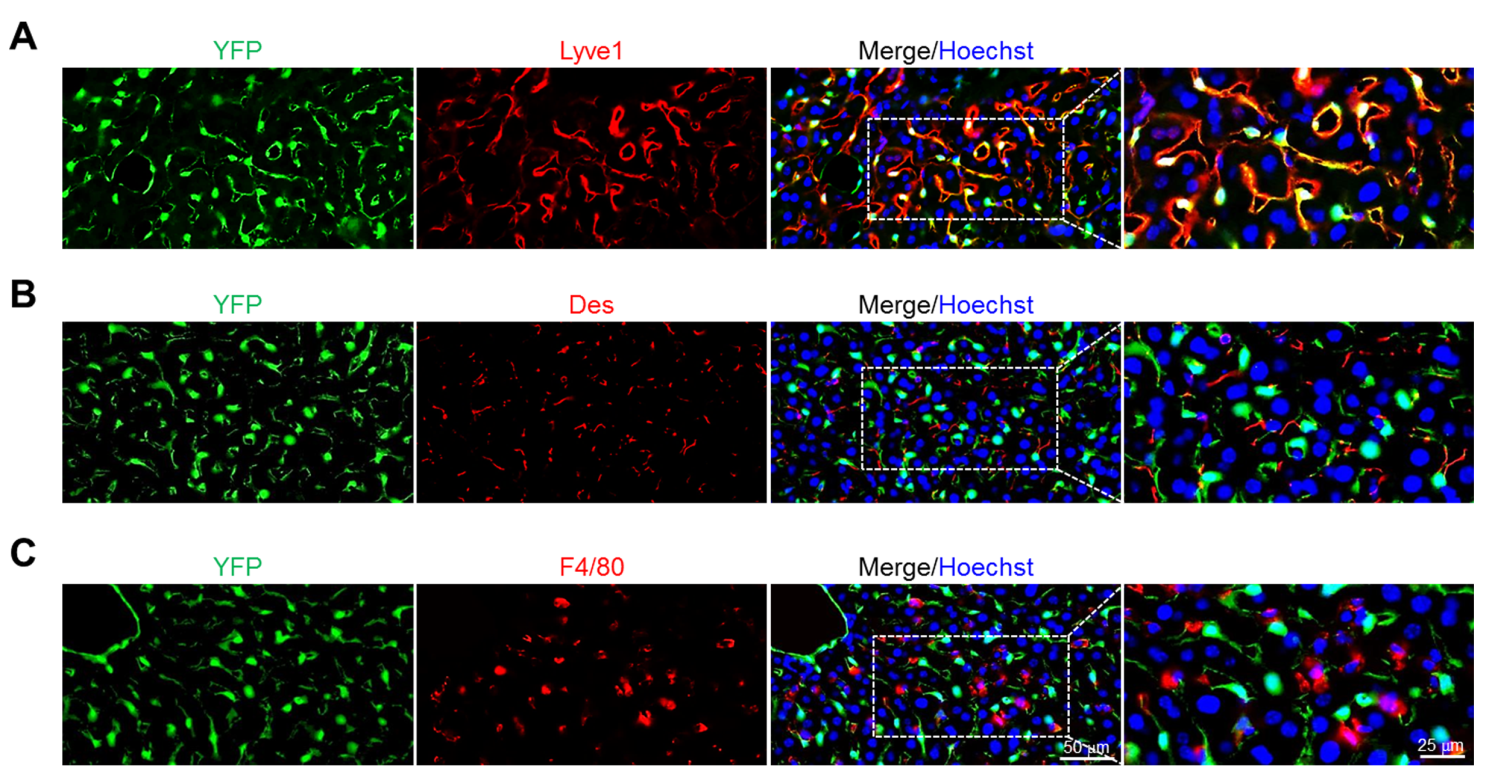


**Supplementary Figure 3.** CDH5-Cre efficiently labels LSECs. **(A)** Co-localization of CDH5-Cre-induced YFP expression and Lyve1 (LSEC marker) was determined in untreated livers using confocal microscopy (n = 3). **(B-C)** Co-localization of CDH5-Cre induced YFP expression and Desmin (HSC marker), F4/80 (macrophage marker) was determined by confocal microscopy in untreated mice (n = 3).


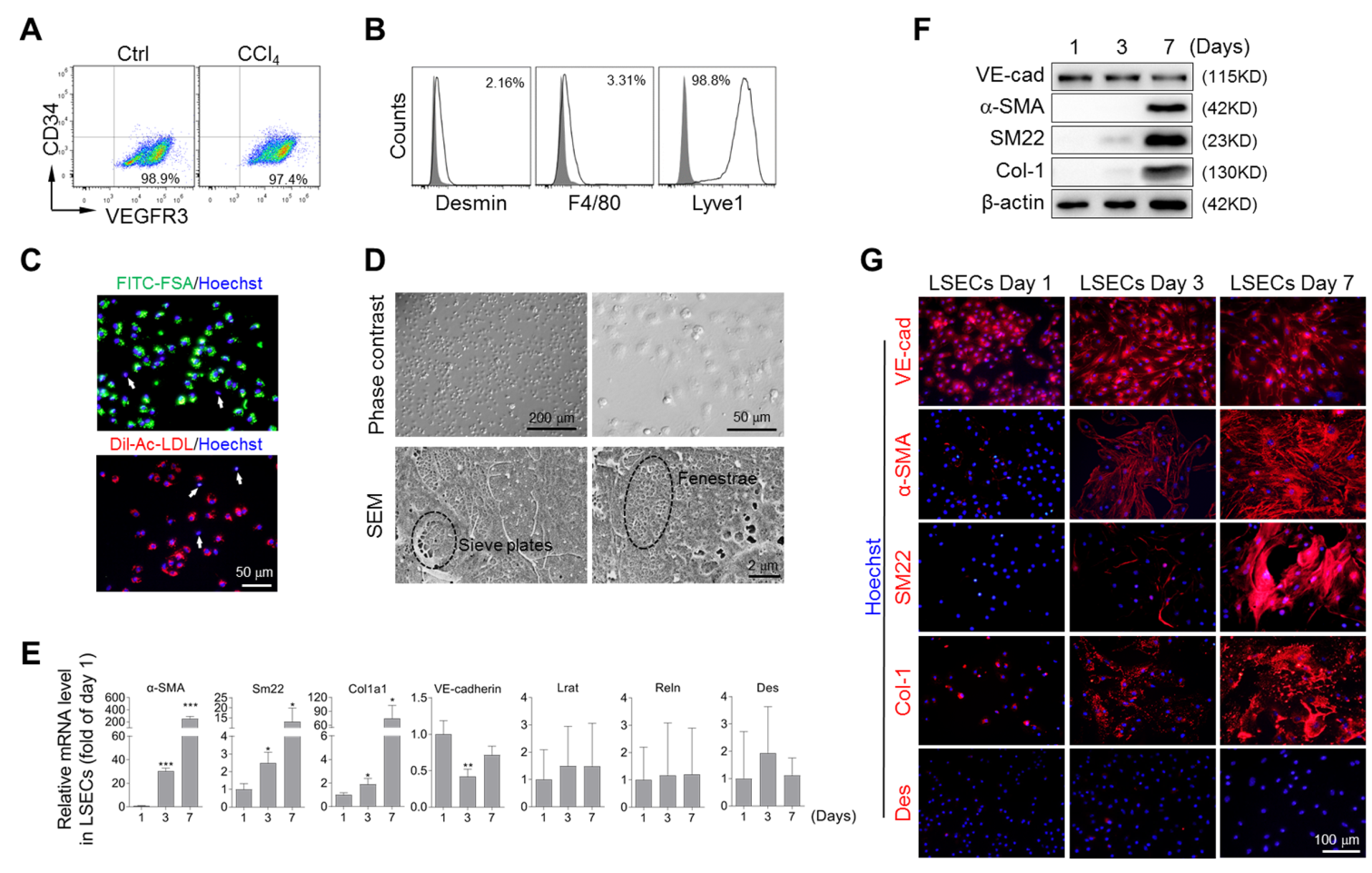


**Supplementary Figure 4.** Isolation and culture of murine LSECs. **(A**,**B)** LSECs were isolated from C57BL/6 mice and analyzed by FACS after staining with VEGFR3 and CD34 (A), or Desmin, F4/80, and Lyve1 (B). **(C)** Freshly isolated LSECs were seeded in dishes, and incubated with FITC-FSA (upper) or Dil-Ac-LDL (lower) and counterstained with Hoechst. White arrowheads indicate LSECs that are negative for uptake of FITC-FSA or Dil-Ac-LDL, respectively. **(D)** LSECs were cultured for 24 h, and observed under a microscope or SEM. Fenestrae and sieve plates are indicated. **(E-G)** LSECs from normal mice were cultured in vitro for 1, 3 and 7 days and then evaluated by qPCR (E, *P < 0.05, **P < 0.01, ***P < 0.001 *vs* Day 1, using two-tail *t* test), western-blot (F) and immunofluorescence (G) for VE-cadherin, mesenchymal genes (α-SMA, SM22 and Col-1) and HSC markers (Lrat, Reln and Des).


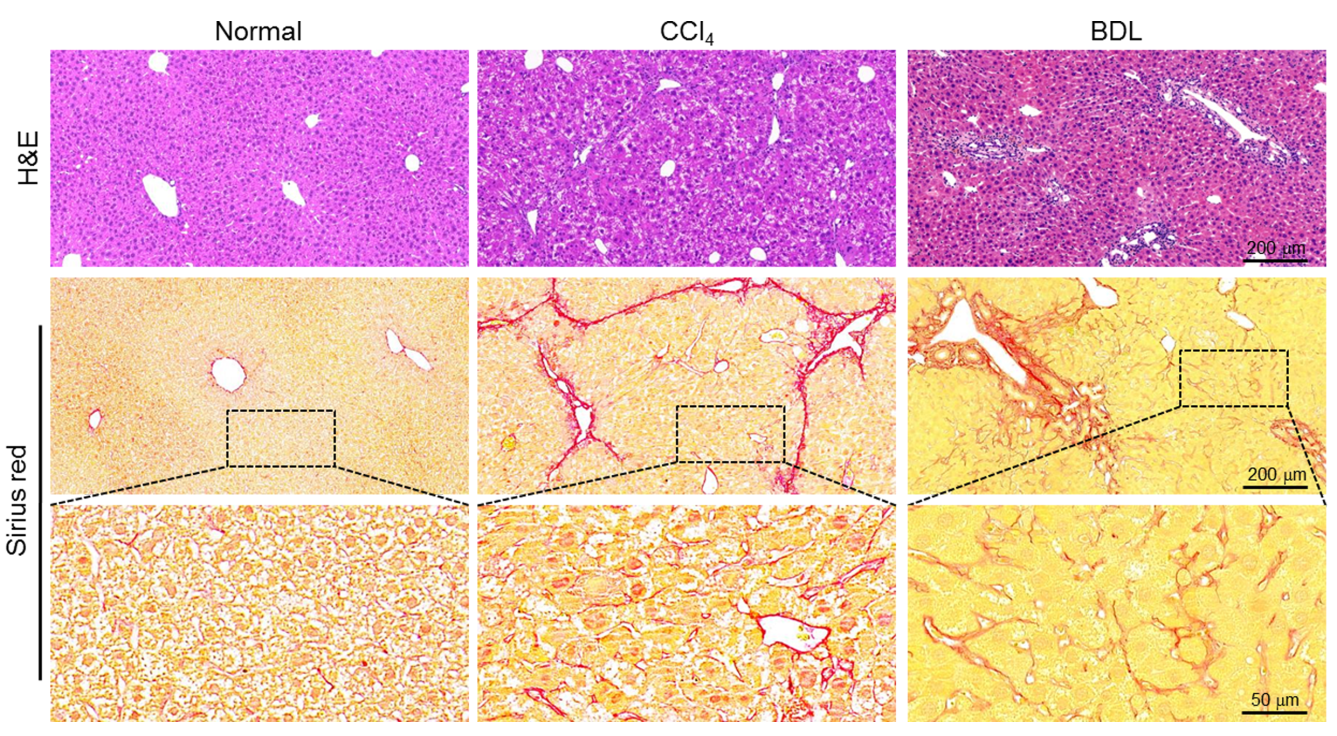


**Supplementary Figure 5.** Establishment of CCl_4_- and BDL-induced liver fibrosis mouse models. Liver samples from the normal and CCl_4_ or BDL injured mice were collected and stained with H&E and sirius red, respectively. Sinusoidal fibrosis emerged in the fibrotic liver sections was representatively shown (bottom enlarged insets).


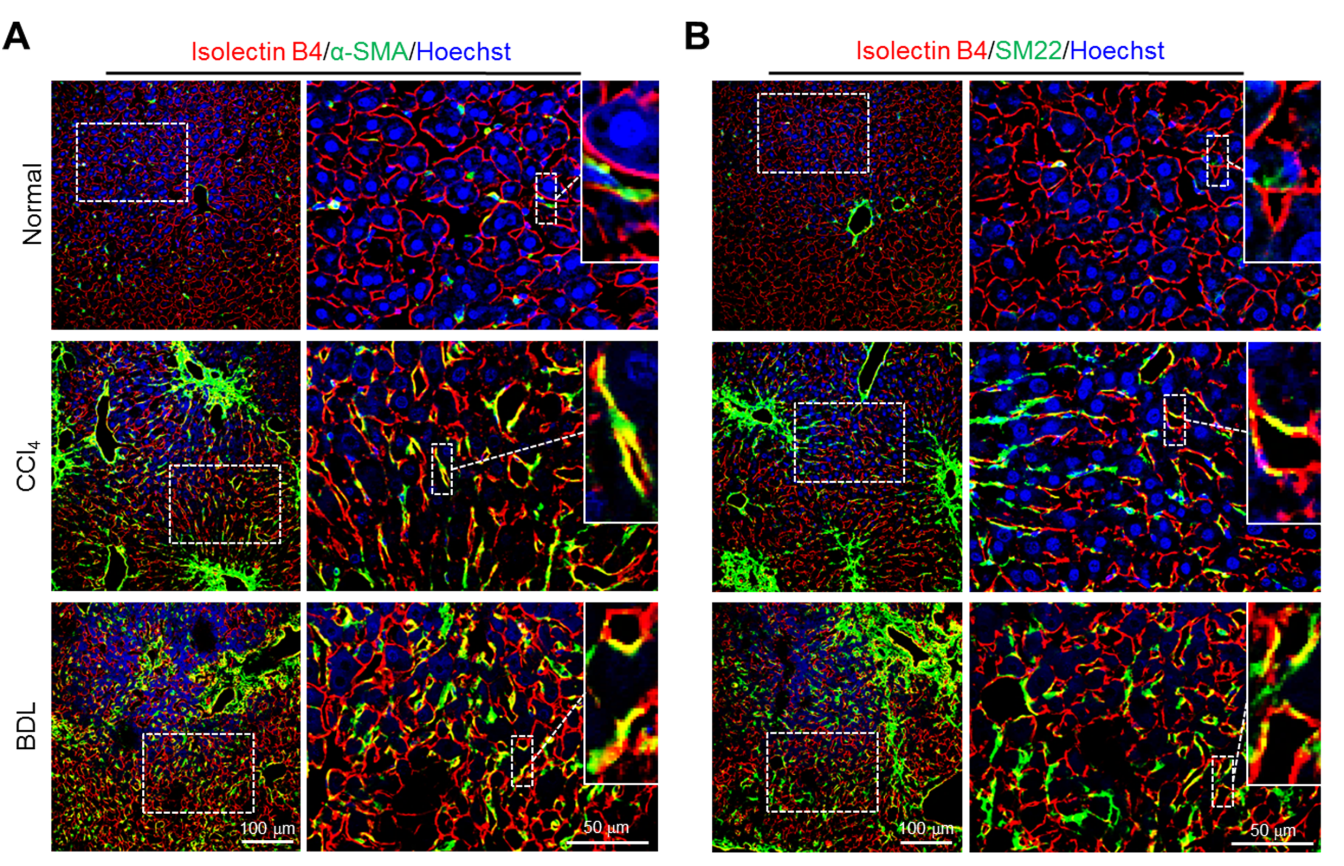


**Supplementary Figure 6.** Colocalization of LSECs and mesenchymal markers in mice with CCl_4_- and BDL-fibrosis. **(A**,**B)** Liver sections from normal and CCl_4_- or BDL-induced fibrotic mice were stained with Isolectin B4 plus α-SMA (A) or SM22 (B). The representative perisinusoidal areas (box with dotted lines) were shown at higher magnifications. Typical cells double-stained with Isolectin B4 and α-SMA or SM22 were enlarged (insets).

## Supplementary Tables

**Supplementary Table S1.** Clinical data of healthy and cirrhotic liver samples from patients.

| **No.** | **Sex** | **Age** | **Grade and Stage** | **Pathogenesis** |
| --- | --- | --- | --- | --- |
| 1 | F | 38 | / | hepatic hemangioma |
| 2 | M | 42 | / | hepatic hemangioma |
| 3 | F | 37 | / | hepatic hemangioma |
| 4 | M | 53 | G4S4 | HBV infection |
| 5 | M | 75 | G3S4 | HBV infection |
| 6 | F | 51 | G4S4 | HBV infection |
| 7 | F | 52 | G4S3 | HBV infection |
| 8 | M | 43 | G2S4 | HBV infection |
| 9 | M | 45 | G2S3 | HBV infection |
| 10 | M | 64 | G3S4 | HBV infection |
| 11 | M | 54 | G2S4 | HBV infection |

**Supplementary Table S2.** Antibodies used in this study.

| **Antigens** | **Catalogue No.** | **Purpose (Dilution)** | **Suppliers** |
| --- | --- | --- | --- |
| Collagen I | ab34710 | IF(1:300) WB(1:500) | Abcam |
| α-SMA | ab124964 | IF(1:300) WB(1:1000) FACS(1:100) | Abcam |
| SM22 | ab14106 | IF(1:200) WB(1:1000) FACS(1:100) | Abcam |
| Isolectin B4 | L-1104 | IF(1:200) | Vector Lab |
| mouse Lyve-1 | 103-M130 | IF(1 ug ml^﹣1^) FACS(2 ug ml^﹣1^) | Relia-Tech |
| human Lyve-1 | 101-M130 | IF(1 ug ml^﹣1^) | Relia-Tech |
| YFP | ab6673 | IF(1:200) | Abcam |
| Desmin | ab32362 | IF(1:200) FACS(1:100) | Abcam |
| VEGFR3 | AF743 | FACS(2 ug ml^﹣1^) | R&D System |
| VE-Cadherin | ab33168 | WB(1:1000) IF(1:200) | Abcam |
| F4/80 | 14-4801 | IF(1:200) | eBioscience |
| FITC-F4/80 | 11-4801 | FACS(1:100) | eBioscience |
| eFluro660-CD34 | 85-50-0341 | FACS(1:100) | eBioscience |
| β-actin | A5441 | WB(1:2000) | Sigma-Aldrich |
| Cy3-anti Rabbit IgG | 111-165-144 | IF(2.5 ug ml^﹣1^) FACS(1.5 ug ml^﹣1^) | Jackson ImmunoResearch |
| Alexa 488-anti Rabbit IgG | A-21206 | IF(1:300) | Invitrogen |
| FITC-anti Rat IgG | 112-095-003 | IF(2.5 ug ml^﹣1^) FACS(1.5 ug ml^﹣1^) | Jackson ImmunoResearch |
| Alexa 594-anti Rat IgG | A-21209 | IF(1:300) | Invitrogen |
| Alexa 488-anti Mouse IgG | A-21202 | IF(1:300) | Invitrogen |
| HRP-anti Rabbit IgG | CW0103 | WB(1:3000) | CoWin Biosciences |
| HRP-anti Mouse IgG | CW0102 | WB(1:3000) | CoWin Biosciences |

**Supplementary Table S3.** Primers used for RT-PCR in this study.

| **Genes** | **Forward (5’-3’)** | **Reverse (5’-3’)** |
| --- | --- | --- |
| β-actin | 5’-GGCTGTATTCCCCTCCATCG | 5’-CCAGTTGGTAACAATGCCATGT |
| α-SMA | 5’-CCCAGACATCAGGGAGTAATGG | 5’-TCTATCGGATACTTCAGCGTCA |
| SM22 | 5’-CCAACAAGGGTCCATCCTACG | 5’-ATCTGGGCGGCCTACATCA |
| Col I | 5’-GCTCCTCTTAGGGGCCACT | 5’-ATTGGGGACCCTTAGGCCAT |
| Snail | 5’-CACACGCTGCCTTGTGTCT | 5’-GGTCAGCAAAAGCACGGTT |
| Slug | 5’-CAGCGAACTGGACACACACA | 5’-ATAGGGCTGTATGCTCCCGAG |
| Twist | 5’-GGACAAGCTGAGCAAGATTCA | 5’-CGGAGAAGGCGTAGCTGAG |
| Zeb1 | 5’-ACCGCCGTCATTTATCCTGAG | 5’-CATCTGGTGTTCCGTTTTCATCA |
| Zeb2 | 5’-AAACGTGGTGAACTATGACAACG | 5’-CTTGCAGAATCTCGCCACTG |
| VE-cad | 5’-CCACTGCTTTGGGAGCCTT | 5’-GGCAGGTAGCATGTTGGGG |
| Lrat | 5’-CCGTCCCTATGAAATCAGCTC | 5’-ATGGGCGACACGGTTTTCC |
| Reln | 5’-GTCATTCCTGGAGCCACAGATC | 5’-GGACATCATCCAAAGCCCACTG |
| Des | 5’-GTGGATGCAGCCACTCTAGC | 5’-TTAGCCGCGATGGTCTCATAC |
